# Supplementary material for: Prospective observational study of peripheral intravenous cannula utilisation and frequency of intravenous fluid delivery in the emergency department—Convenience or necessity?
Source: PLoS One. 2024 Jun 14;19(6):e0305276. doi: 10.1371/journal.pone.0305276 (PMC11178163; doi:10.1371/journal.pone.0305276)
Supplement: Attachment — Submitted filename: Response to reviewers.docx [file pone.0305276.s002.docx]

Emergency Department

The Royal London Hospital, Barts Health NHS Trust

London, England

E1 1BB

+44 7800 948 068

m.willis@doctors.org.uk

Dr Ochuwa Adiketu Babah

Academic Editor

PLOS ONE – Public Library of Science

16 July 2023

Dear Dr Babah,

We thank you for your consideration and review of the original research article entitled ***“Prospective observational study of peripheral intravenous cannula utilisation and frequency of intravenous fluid delivery in the emergency department – convenience or necessity?”***

Please see below a response to the minor corrections requested:

1. Data availability - Please state where (that is, in what database) or from whom the data analyzed for this study can be retrieved.

**Response: the data can be requested from myself (Dr Michael Willis), in a Microsoft Excel format.**

2. Insert a title for figure 1.

**Response: A title is present on lines 142 and 143 for figure 1. Please let me know if this needs to be rectified/in a different formatting.**

3. References - You used et al after listing only one author for most of your citations. With Vancouvre referencing the norm is to list six authors putting et al. Please review the references according.

**Response: this has been rectified in the references section**

Thank you once again for your time in considering this work.

Yours sincerely,

Dr Michael Willis

BSc, MBBS, MRCPCH

Supervisor: Professor Tim Harris

Barts and The London School of Medicine and Dentistry

Queen Mary, University of London

London

England

E1 2AD

+44 207 882 5555

[t.harris@qmul.ac.uk](mailto:t.harris@qmul.ac.uk)
