# Supplementary material for: Prospective observational study of peripheral intravenous cannula utilisation and frequency of intravenous fluid delivery in the emergency department—Convenience or necessity?
Source: PLoS One. 2024 Jun 14;19(6):e0305276. doi: 10.1371/journal.pone.0305276 (PMC11178163; doi:10.1371/journal.pone.0305276)
Supplement: Attachment — Submitted filename: Response to reviewers.docx [file pone.0305276.s003.docx]

Emergency Department

The Royal London Hospital, Barts Health NHS Trust

London, England

E1 1BB

+44 7800 948 068

m.willis@doctors.org.uk

Dr Amit Bahl

Academic Editor

PLOS ONE – Public Library of Science

14 January 2024

Dear Dr Bahl,

We thank you and the reviewers for the considered comments and review of the original research article entitled ***“Prospective observational study of peripheral intravenous cannula utilisation and frequency of intravenous fluid delivery in the emergency department – convenience or necessity?”***

Please see below our responses to the reviewers comments, please note the line references in our responses are for the document entitled “Manuscript”:

**Reviewer #1
Introduction**

- *Suggest re-structuring the introduction- 4th paragraph can be integrated with the first, then follow with risks associated with PIVC and then discuss unused/unnecessary PIVC.*
  - With thanks. We have restructured the paragraph as suggested
- *Authors could also consider discussing the environmental impact of the inappropriate use of PIVC*
  - We could not find studies comparing simple phlebotomy vs PIVC insertion and the environmental impact of this. Therefore we have added a line (76-78) to reflect this important point.
- *Line 63: where were the other studies conducted?*
  - Webster et al. 2019 is a Cochrane review which included 7 trials. 6 were single centre, and one was a multi-centre trial. One trial was based in India, one in the UK, and the rest in Australia. 6 were based in acute inpatient settings, and one in a community hospital setting. Maki et al. 2016 included 20 prospective studies in their systematic review observing blood stream infection from PIVCs. 7 studies were based on medical inpatient wards, 7 on surgical wards, 2 in ITU and 4 in haematology/oncology wards. We have added a line (70-71) to reflect this.
- *Lines 63-65- the sentence related to OR is not quite clear: are you saying that the risk of catheter associated infections is 6 times greater when inserted in the ED compared to inpatient wards? What is the p value? I’m not sure what the OR of 2.3 is referring to. Please also include where this study was conducted and if it was a large or small study eg. Are these results from a single centre ED in X country?*
  - The study Trinhh et al. 2011 was a single tertiary centre hospital in the US. It was a retrospective case note review over a 3 year period. The authors identified a total of 544 staphylococcus aureus bacteraemias, of which 24 were identified to be caused by the PIVC through clinical signs (phlebitis around the site) and positive tip cultures of the device. 16 of the PIVC-related S. aureus bacteremias (67%), the PIVC was placed in the emergency department, 4 (17%) were placed in an inpatient unit, 2 (8%) were placed by emergency medical services prior to admission, and 2 (8%) were placed at outside hospitals. The risk is 6 times greater for cannulas inserted in the ED (OR 6.03, p< 0.001) compared with those inserted as an inpatient (0.17, p<0.001). Note the previous reported OR of 0.23 was a mistake. The total were compared with the total PIVCs observed prospectively over a one day period (317 total PIVCs in situ with 79 inserted in A&E and 170 inserted on the ward). Note the confidence intervals are reported incorrectly for the OR of PIVCs inserted in the emergency department, with a typo at the upper bound. We could not find a revision submitted by the authors. The Fischer’s exact test is <0.001 for both. I have included the table from the paper for reference.
  - We have updated the introduction to reflect these points in lines 72-73

- *What is the rational for focusing only on the use of PIVC for IV fluids? What about unnecessary use related to medication? Or generally on the appropriate use of PIVC?*
  - There is previous evidence to show IV fluids were being utilised for inappropriate indications in the emergency department, examples include in renal colic and alcohol intoxication where IV fluids are frequently prescribed despite data showing no changes in outcomes. They are also the most common intervention given via a PIVC secondary to usage for phlebotomy. We felt it was more appropriate to focus on inappropriate IV fluid usage as we could formulate *a priori* indications related to known Trust guidelines. Given the heterogeneity and number of different medications given in a tertiary ED we felt it would be difficult to introduce *a priori* indications for this category. It is also hard to define appropriate vs inappropriate medications around delivering these as IV or PO. We could use guidelines but there are many good reasons the treating team may have deviated from these which were not apparent to the researchers. Furthermore the main data collectors were medical students in their 3^rd^-4^th^ year of study, who would not be able to clinically determine every medication’s indication and if this was suitably given without *a priori* indications.
- *The first part of the aim isn’t quite clear- are you assessing the prevalence of PIVC use in the ED or are you describing the indications for PIVC use in the ED?*
  - The first aim is to describe both the prevalence and their usage. This has been clarified in lines 80-81. The secondary aim was to assess the indications for IV fluid usage, and categorise PIVCs in to used, unused, and unnecessary.

**Methods**

- *Need to specifically say that the study site were ED centers*
  - This is described in the introduction in lines 80-81, we have also added a clarification point in line 87 in the methods.
- *Suggest providing a brief description of the ED sites- size, volume of patients, staffing, training, types of patients*
  - We have updated the methodology to reflect this in lines 87-90
- *Selection of participants might be better presented as inclusion and exclusion criteria. Unlikely that all patients would have been followed given the design used. Rather the inclusion criteria would have been adult patients, presenting to the ED with X conditions during the data collection periods and who were ?discharged from the ED by the end of the data collection period?*
  - This has been clarified in lines 102-106.
- *Why were patients with minor injuries excluded?*
  - The treatment of patients in minor injuries typically did not involve inserting a PIVC. If patients required IV medications or fluid, they were usually transferred into majors and would have been captured by our inclusion criteria with a PIVC generally inserted in majors.
- *Was a data collection sheet used? If so, could you provide it?*
  - Data was collected using an electronic tablet on an online spreadsheet. The categories recorded are included in lines 116-117.
- *Was the date of PIVC insertion recorded?*
  - Yes, this is detailed in lines 119-123. The date and time of PIVC insertion, and date and time of patient discharge was recorded.
- *Were the indications for PIVC used recorded? This isn't actually stated in the methods. Were pre-determined categories used?*
  - The only indications were the *a priori* indications which are listed in lines 125-129. We did not otherwise record PIVC indications.
- *Were the diagnoses/likely diagnoses for the included patients recorded?*
  - No, only the *a priori* indications for IV fluid usage.
- *What was the length of stay for the included patients?*
  - We have not included this in our analysis, however it would have been a maximum of 14 hours in Centre A or 6 hours in Centre B (if patients stayed longer they would have been excluded as part of our study recruitment exclusions)
- *IV medications is listed as one of the indications for giving IV fluids- Have you classified IV medication as IV fluids? Please clarify*
  - Some IV medications/contrast/blood products require an IV “push” of fluid. This was included in the *a priori* exclusions as the IV fluid is needed as part of administering medications. We have clarified this point in the methodology lines 126-127.
- *Authors state that patients were included if in a non-resus area and excluded if transferred to a resus area- fluid resuscitation is listed as one of the indications for IV fluids- seems to clash with the inclusion/exclusion criteria- authors please clarify*
  - Some patients in the “majors” area were given IV fluid resuscitation if the resus area was full, or were not critically unwell enough to warrant transfer as decided by a senior clinician. This point captured those patients.
- *How did the team determine the indications for the administration of fluids? Did they ask the clinicians managing the patient or check the patient notes? (noted that data was collected using patient observations, records and clinicians but it would be good to specifically say how the indications for use were determined)*
  - As noted in lines 117-119 “Data concerning PIVC insertion and utilisation were prospectively recorded from patient arrival until discharge from the ED through direct observation, prospective ED record review and/or clarification with the patient’s clinician”
  - The specific indications are as described in the *“a priori”* indication list.
- *Definitions of unnecessary and necessary use of PIVC should be presented earlier*
  - This has been moved to earlier in the methodology section

**Results**

- *Definitions for the categories should be presented in the methods not the results – the ‘unused and necessary’ category was not previously mentioned or defined*
  - Definitions are listed in the methodology and we have included a definition for “unused” PIVCs, and we have felt that repeating the definitions in the results table provides clarity to those who may not read the full paper.

- *Would ‘unused’ PIVC also be considered ‘unnecessary’?*
  - Yes, but we wanted to distinguish those unnecessary PIVCs that were utilised inappropriately for IV fluid administration, therefore we have used both unused and unnecessary
- *Table 2- were these categories pre-determined? It is not uncommon in an ED setting to place an IV in the event the patient will be admitted or deteriorates- was that indication included in the data collection?*
  - We acknowledge the argument for ‘just in case PIVCs’. However predicting clinical risk of deterioration is challenging and risks many PIVCs being placed per use and may be regarded as wasteful. We have noted patients who had a PIVC inserted, and who subsequently deteriorated requiring admission to a resuscitation area as very low (see discussion). We did not recruit patients in the resuscitation area who are at higher risk in part for this reason. Liberal placement of PIVCs may also promote inappropriate use of IV fluids. In addition PIVCs take longer to insert, and have a higher environmental impact. There was otherwise no indication in the data collection for “just in case”

**Discussion**

- *This could be more concise, The numerous subheadings are not necessary for this study. Focus on what you found and possible explanations for the findings. The ‘just in case’ category was not noted as in indication in the current study- is it that there were no ‘just in case’ scenarios or was this not collected?*
  - We have rewritten the discussion to be more concise, removed sections that do not explicitly relate to our results, and removed the subheadings. We would appreciate the reviewers’ opinion on removing the subheadings, as we feel this has now reduced some of the structure and clarity in the discussion.
  - The “just in case” category was not included as an indication (see above answer). We have demonstrated that 11 patients with a PIVC were transferred to resus, and utilised this information to inform further discussion around the need for “just in case” PIVCs
- *The study was conducted in two countries- someone discussion around similarities/differences would be useful.*
  - We discuss the similarities in the methodology section, but have chosen not to analyse the population differences between the two centres as this study was not powered or designed to be a comparison between two different hospitals. We have added lines in the discussion around some of the differences (159-163)

**Reviewer #2:**

- *This paper requires edits prior to publication. Firstly, it is not the largest study to examine the utilisation of PIVC within the ED setting: Hawkins T et al. Peripheral Intravenous Cannula Insertion and Use in the Emergency Department: An Intervention Study. Acad Emerg Med. 2018 Jan;25(1):26-32*.
  - This is the largest *international* prospective study to date. It was not single centre, and we wanted to reflect this. However we can understand this line may be misleading, and so we have edited this in lines 153-154
- *Secondly, an explanation as to why the two sites were chosen as comparison or similar sites is required. More detail into the methods as to why differing time frames, sampling methods and data collection processes were used is needed to strengthen the study. The differences are a significant risk to the methods and need to be explained.*
  - These are well made points. The data collection primarily reflected the availability of the research team. The study was not funded and all researchers completed training and data collection in their own time which was limited by their own work and study. The two sites were chosen as there were established academic links between them and existing research infrastructure, so making roll out of the study more likely to succeed. There is explanation in the limitations (lines 236-237) that describe the reasons for the differing time frames and sampling methods were used – due to data collector availability. We are happy to be advised if the reviewer suggests additional or alternative wording.
  - We have added a section around the main differences in the hospitals in the discussion.
- *Additional explanation as to the data analysis methods used would be of benefit including reworking the tables as in the current format the results are unclear*.
  - Thank you. We have reformatted the tables to provide more clarity with the results and more consistency with matching definitions.

Thank you once again for your time in considering this work.

Yours sincerely,

Dr Michael Willis

BSc, MBBS, MRCPCH

Supervisor: Professor Tim Harris

Barts and The London School of Medicine and Dentistry

Queen Mary, University of London

London

England

E1 2AD

+44 207 882 5555

[t.harris@qmul.ac.uk](mailto:t.harris@qmul.ac.uk)
