# Supplementary material for: Prospective observational study of peripheral intravenous cannula utilisation and frequency of intravenous fluid delivery in the emergency department—Convenience or necessity?
Source: PLoS One. 2024 Jun 14;19(6):e0305276. doi: 10.1371/journal.pone.0305276 (PMC11178163; doi:10.1371/journal.pone.0305276)
Supplement: Attachment — Submitted filename: Response to reviewers.docx [file pone.0305276.s004.docx]

Emergency Department

The Royal London Hospital, Barts Health NHS Trust

London, England

E1 1BB

+44 7800 948 068

m.willis@doctors.org.uk

Dr Robert Jeenchen Chen

Academic Editor

PLOS ONE – Public Library of Science

1 May 2024

Dear Dr Chen,

We thank you and the reviewers for the considered comments and review of the original research article entitled ***“Prospective observational study of peripheral intravenous cannula utilisation and frequency of intravenous fluid delivery in the emergency department – convenience or necessity?”***

Please see below our responses to the reviewers comments, please note the line references in our responses are for the document entitled “Manuscript”:

**Reviewer #1**

- *Thanks for addressing the comments. The only minor comment is if the authors could include how the criteria for administration of IVF was determined. Is it based on existing guidance or was it developed by the authors/expert team for the study*
  - These criteria were based on existing NICE (National Institute for Health and Clinical Excellence) guidance on indications for IV fluid in adults, we have clarified and referenced this in lines 127-127.

**Reviewer #2**

- *Thank you for your edits to date. I would suggest the following minor edits prior to publication- Line 71- please expand ITU to explain the term.*
  - We have adjusted “ITU” to “ICU” Intensive Care Unit as this is a more internationally recognised term in line 71
- *Line 159- should be 'centre' not 'centres'.*
  - This has been edited.
- *Line 175- your results conflict with this study, in which the authors accept PIVC utilisation for IV fluids as an acceptable reason for PIVC insertion. I suggest you link this to your paragraph (line 191) which explains this further.*
  - We have moved this section on Hawkins et al. 2017 to lines 188-192, linking the distinction our work has made to identifying IV fluid treatment with *a priori* criteria.
- *In the current format it does not flow (ie move the paragraph involving lines 179-189 down further to line 207).*
  - We agree and have moved the paragraph at your suggestion.

Thank you once again for your time in considering this work.

Yours sincerely,

Dr Michael Willis

BSc, MBBS, MRCPCH

Supervisor: Professor Tim Harris

Barts and The London School of Medicine and Dentistry

Queen Mary, University of London

London

England

E1 2AD

+44 207 882 5555

[t.harris@qmul.ac.uk](mailto:t.harris@qmul.ac.uk)
